# Supplementary material for: Comparative multiomics analysis of cell physiological state after culture in a basket bioreactor
Source: Sci Rep. 2022 Nov 23;12:20161. doi: 10.1038/s41598-022-24687-4 (PMC9686226; doi:10.1038/s41598-022-24687-4)
Supplement: Supplementary file 1 — Supplementary Information 1. [file 41598_2022_24687_MOESM1_ESM.zip › raw data/Transcriptomic raw data/7.AS/2.dasplot/TE1vsTC1/RI/20_LOC103236249_NW_023666037.1_54580878_54581854_+@NW_023666037.1_54580878_54583037_+@NW_023666037.1_54582849_54583037_+.pdf]

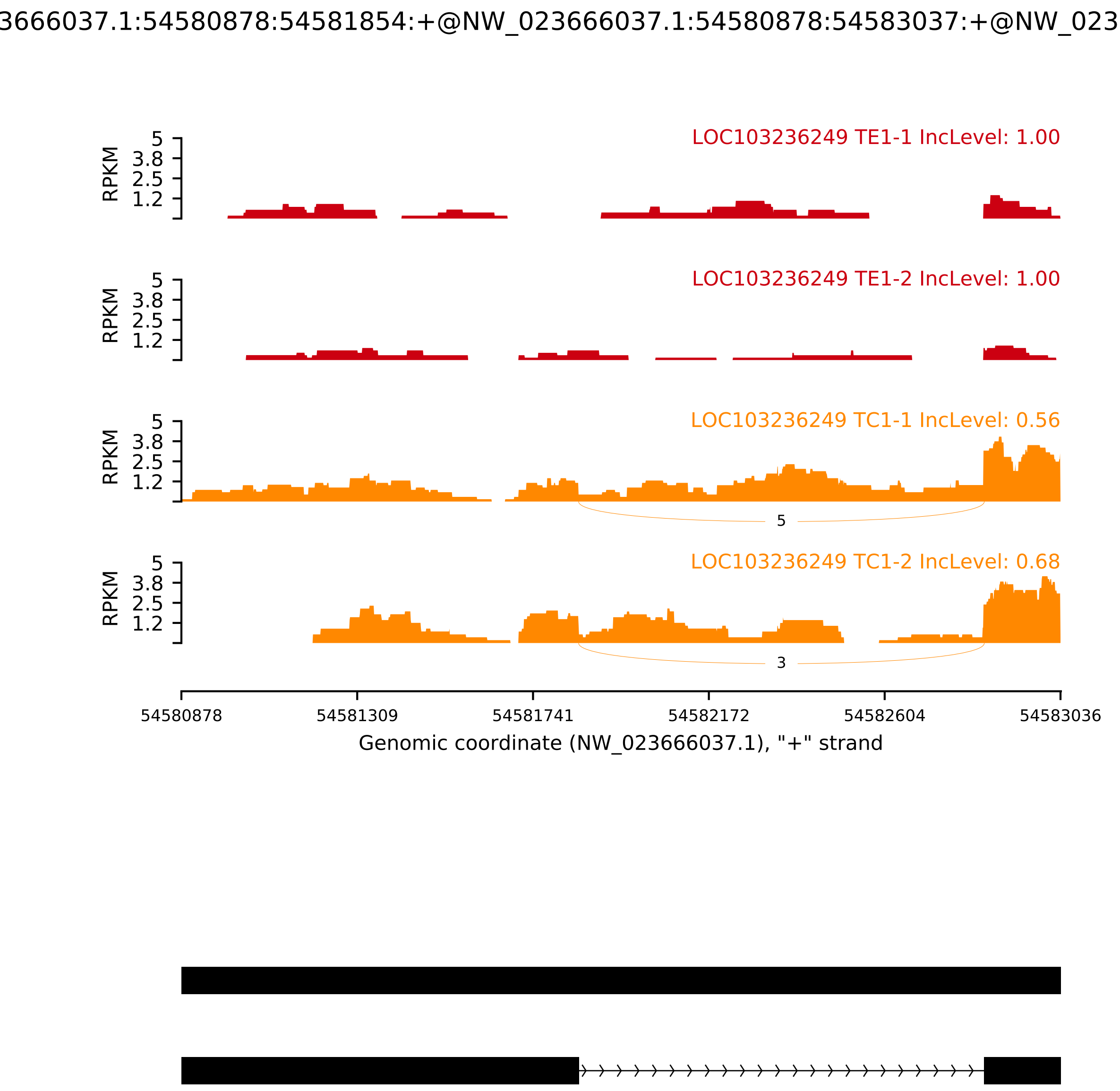
3666037.1:54580878:54581854:+@NW_023666037.1:54580878:54583037:+@NW_023666037.1:54582849:5458
LOC103236249 TE1-1 IncLevel: 1.00
LOC103236249 TE1-2 IncLevel: 1.00
LOC103236249 TC1-1 IncLevel: 0.56
LOC103236249 TC1-2 IncLevel: 0.68
RPKM
5
3.8
2.5
1.2
5
3
54580878
54581309
54581741
54582172
54582604
54583036
Genomic coordinate (NW_023666037.1), "+" strand
